# Theoretical Study on the High Polymer Molecular Weight of Heteroatom-Substituted Constrained Geometry Catalyst

**DOI:** 10.3390/polym16233251

**Published:** 2024-11-22

**Authors:** Xinyue Du, Congjing Ren, Xiaodong Hong, Jingdai Wang, Yongrong Yang, Zuwei Liao

**Affiliations:** 1 State Key Laboratory of Chemical Engineering, Department of Chemical and Biological Engineering, Zhejiang University, Hangzhou 310058, China; 22128049@zju.edu.cn (X.D.); yangyr@zju.edu.cn (Y.Y.); liaozw@zju.edu.cn (Z.L.); 2 Ningbo Innovation Center, Zhejiang University, Ningbo 315100, China; 3 Engineering Research Center of Functional Materials Intelligent Manufacturing of Zhejiang Province, ZJU-Hangzhou Global Scientific and Technological Innovation Center, Zhejiang University, Hangzhou 311215, China

**Keywords:** constrained geometry catalysts, polymerization, quantitative structure–property relationship

## Abstract

This theoretical study investigates the high molecular weight (Mw) production in copolymerization of ethylene and 1-octene using heteroatom-substituted constrained geometry catalysts (CGCs). The research explores the correlation between chain termination reactions and polymer molecular weight, revealing that the Gibbs free energy barrier of the chain termination reactions is positively linked to the molecular weight. Quantitative structure–property relationship models were constructed, indicating that molecular descriptors such as atom charge, orbital energy, and buried volume significantly influence the polymer molecular weight.

## 1. Introduction

**Scheme 1:**
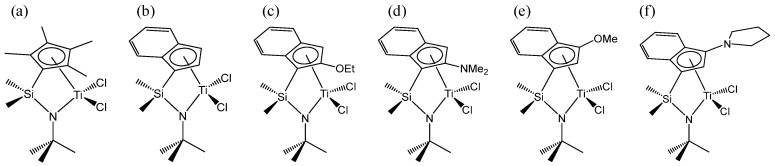
Schematic representation of heteroatom-substituted constrained geometry complexes. (**a**) 1-C_5_Me_4_, (**b**) 2-Ind, (**c**) 3-OEt, (**d**) 4-NMe_2_, (**e**) 5-OMe, (**f**) 6-NC_4_H_8_.

Catalysts of the transition metals in Group IV are widely used in the polymerization of ethylene and α-olefins. With the rapid development and application of polyolefin elastomers such as polyolefin elastomer (POE) and olefin block copolymer (OBC), the copolymerization of ethylene and α-olefins has attracted extensive research interest [1,2,3,4,5]. Constrained geometry catalyst (CGC) is widely used in the polymerization of ethylene and α-olefins due to its excellent copolymerization ability. The design of CGC uses an amino group to replace one of the cyclopentadienyl rings in the traditional metallocene catalyst and connects them through a bridge group, forming a sandwich structure with a small angle [6]. The typical CGC structure is shown in Scheme 1a. The unique sandwich structure of CGC makes the space around the central metal more open, with a high α-olefin insertion rate, narrow molecular weight distribution, and good thermal stability. Research on CGC-related work has been continuously reported [7,8,9,10,11,12].

The alteration of catalyst structure determines the difference in catalytic performance. Earlier, Stevens [13] investigated the structure–activity relationship of CGC, discussing the correlation between the catalytic performance and four types of CGCs with different structural characteristics: ring structures (C_5_Me_4_, C_5_H_4_ and Indenyl), substituents on the N atom (^t^Bu, Ph and 4-F-Ph), bridge types ((SiMe_2_)_2_, SiMe_2_ and (CH_2_)_2_), and central metals (Ti and Zr). The results showed that for the discussed different ring structures and substituents on the N atom, electron-withdrawing groups lead to decreased activity and copolymer monomer insertion rate, while an increase in the electron density of the central metal enhances the catalytic activity. To further increase the electron density of the central metal, Klosin et al. [14,15] studied several amino- and alkoxy-substituted indenyl CGCs, as shown in Scheme 1b–f. This is because alkoxy and amino substituents have been proven to be highly effective electron-donating groups in electrophilic aromatic substitution reactions and substituted ferrocenes. Based on the experimental data of these heteroatom-substituted CGCs catalyzing the copolymerization of ethylene and 1-octene, it can be found that compared to typical CGC catalysts (Scheme 1a), the product molecular weight of these catalysts is significantly increased, and the 3-amino-substituted catalyst exhibits the highest catalytic activity and forms the highest molecular weight ethylene/octene copolymers.

In recent years, computational chemistry has been widely applied to studying catalytic mechanisms and structure–activity relationships of transition metal catalysts [16,17,18,19,20,21]. Ratanasak et al. [22] used density functional theory (DFT) to investigate the olefin polymerization process catalyzed by ansa-Zr catalysts. By calculating the energies of various structures in the reaction pathway, they found a significant correlation between the intrinsic activation energy of the ethylene insertion process and the experimental activity. They constructed quantitative structure–property relationship (QSPR) models of the intrinsic activation energy and molecular descriptors based on this. Zaccaria et al. [23] studied the copolymerization properties of 19 metallocene and post-metallocene catalysts for olefin polymerization. Combining the Curtin–Hammett principle and DFT, they accurately predicted the copolymerization ratio of ethylene and propylene inserted into the metal alkyl chain. The copolymerization ratios obtained by calculating the energy difference between the transition states of the two monomers had high accuracy compared to the experimental values. Subsequently, Maity et al. [24] constructed a QSPR model for these 19 catalysts using DFT and MLR, including electronic and steric descriptors. The model showed that strong electron-withdrawing groups favored the insertion of α-olefins. Uborsky et al. [25] constructed a QSAR model containing 28 C1-symmetric silyl-bridged ansa-Zr catalysts for the copolymerization of ethylene and α-olefins to predict the relevant energy differences describing molecular weight and comonomer affinity. The author optimized the geometric structures of the dichloride precursors of the catalysts using DFT and collected 71 molecular descriptors, including 2D/3D geometric descriptors, charges, and orbital energies. The QSAR model showed that steric effects had a stronger influence on product molecular weight and comonomer affinity than electronic effects, but electronic effects were also not negligible. Li et al. [26] studied the ethylene polymerization process catalyzed by three different ligand half-metallocene titanium catalysts using DFT and constructed energy profiles, including chain initiation, chain propagation, and chain termination. By calculating the reaction energy barriers of chain initiation and chain propagation, it was found that chain initiation had a lower reaction rate than chain propagation, and the ethylene polymerization activity of the three catalysts was consistent with the order of the reaction energy barriers of chain initiation. During the chain termination stage, the molecular weight characteristics of the polymer were studied by considering β-H elimination reactions and β-H transfer to ethylene reactions. Lu et al. [27] combined DFT and multiple linear regression methods to construct energy profiles for Pd(II)-catalyzed ethylene polymerization as an example. Six quantitative structure–energy relationship models were constructed, considering ethylene coordination, insertion, and β-H elimination processes. The models were able to predict the energy profile with high accuracy.

**Scheme 2:**
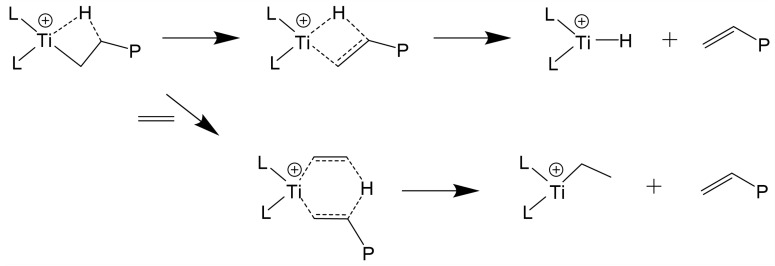
Schematic representation of the β-hydrogen elimination and β-H transfer to monomer of metallocene catalyst.

Previously, we investigated the quantitative structure–property relationship of cyclopentadienyl CGC, analyzing the relationship between the reaction barriers in the chain initiation and chain propagation stages and catalytic activity [28]. In this work, based on the chain termination process, we explored the relationship between the polymer molecular weight and reaction barriers of indenyl CGC substituted with amino and alkoxy groups. The chain termination reactions include β-H elimination and β-H transfer to monomers (Scheme 2), with the chain structures containing different monomers (ethylene and 1-octene).

## 2. Computational Methods

All calculations were performed using the Gaussian 16 program [29]. Geometric optimization was carried out using the B3LYP-D3 functional [30,31,32,33], with the 6-31G** basis set applied to C, H, O, N, and Si atoms, while the LANL2TZ pseudopotential basis set used to metal atoms. Frequency analysis was conducted on all optimized intermediate structures to ensure the absence of imaginary frequencies, while transition state structures were verified to possess only one imaginary frequency. The intrinsic reaction coordinates (IRC) of all transition states were calculated. Single-point calculations were performed using the M06-2X/def2-TZVPP functional and basis set [24,26,34]. Solvent corrections were applied using the polarizable continuum model (PCM) [35], with n-hexane (ε = 1.8819) serving as the solvent. Gibbs free-energy calculations were performed at 413.15 K, and the structures with the lowest transition state energy were selected. Discussion about the impact of the conformation on the chain structure can be found in the Supplementary Materials S1. The tridimensional geometrical structures were visualized using CYLView (Version 1.0) [36]. Buried volumes were calculated with the Sambvca2.1 package [37]. For the construction of QSPR models, a multivariate linear regression analysis was employed.

## 3. Discussion and Results

### 3.1. β-H Elimination

**Figure 1:**
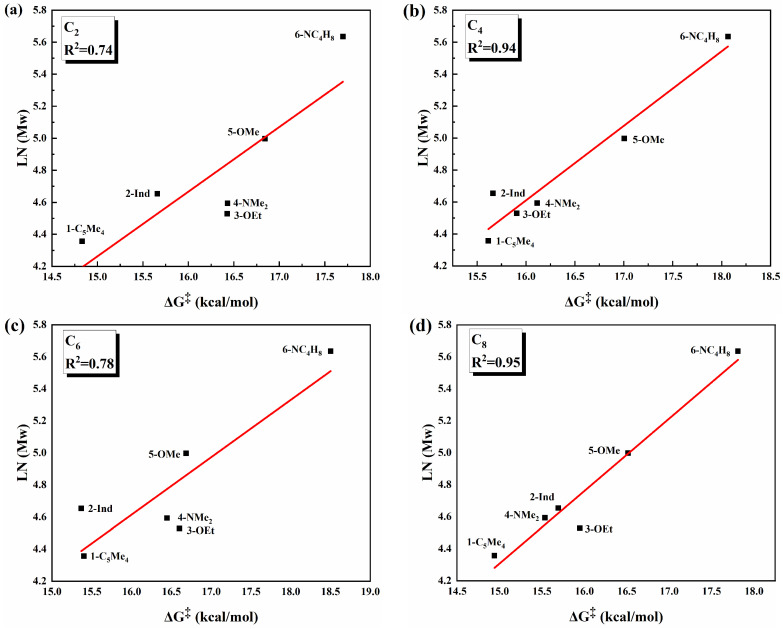
Correlation between the molecular weight (Mw) and energy barriers (∆G^‡^) of β-H elimination for the chain inserted by (**a**) one, (**b**) two, (**c**) three, and (**d**) four ethylene molecules. Molecular weight of polymer is 78 kg/mol by 1-C_5_Me_4_, 105 kg/mol by 2-Ind, 92.7 kg/mol by 3-OEt, 98.8 kg/mol by 4-NMe_2_, 148 kg/mol by 5-OMe, and 280 kg/mol by 6-NC_4_H_8_, respectively.

Firstly, we considered the β-H elimination reaction of the chain structure inserted by ethylene. The Gibbs free energy barriers of β-H elimination of chain structure inserted by one, two, three, and four ethylene molecules were calculated, respectively, which was correlated with the molecular weight of the product, as shown in Figure 1. The calculated results are also shown in Table S5 (in the Supplementary Materials S2). The molecule weight of the product is obtained from Klosin et al. [15]. It can be seen that the molecular weights of polymers by catalysts 5-OMe and 6-NC_4_H_8_ substituted at indene position 3 are higher than those of catalysts 4-NMe_2_ and 3-OEt substituted at indene position 2, and the calculated energy barrier of β-H elimination reaction is correspondingly higher. When tetramethylcyclopentadienyl is used as the ring structure, the molecular weight of the product is the lowest, and the calculated β-H elimination reaction energy barrier is lower than that of indene CGC.

When only the chain structure formed after ethylene molecule insertion is considered, the Gibbs free energy barrier of the β-H elimination reaction is positively correlated with the molecular weight of the product; that is, the higher the molecular weight of the product, the higher the energy barrier to be overcome in the β-H elimination reaction. Among them, the positive correlation between the β-H elimination barrier of the n-amyl chain and the n-nonyl chain structure formed by the insertion of two molecules of ethylene and four molecules of ethylene, and the molecular weight of the product is more significant, and the R^2^ is greater than 0.9. However, due to the limited variety of chain structures considered, no systematic conclusion can be obtained.

**Figure 2:**
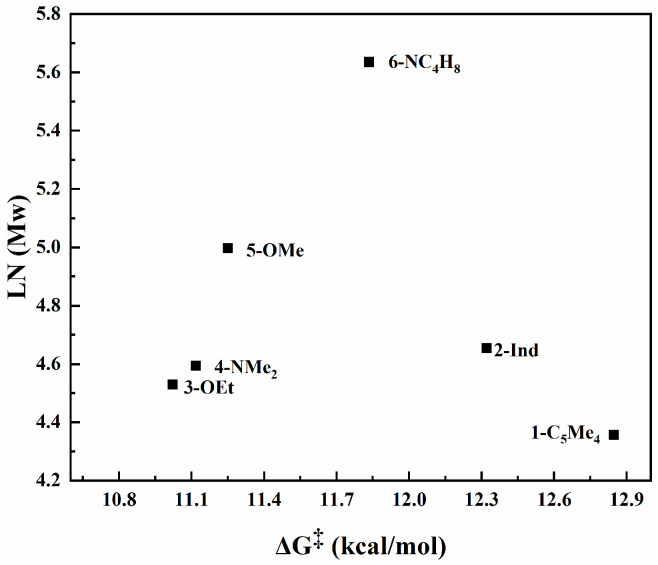
Correlation between the molecular weight (Mw) and energy barriers (∆G^‡^) of β-H elimination for the chain inserted by ethylene and 1-octene. Molecular weight of polymer is 78 kg/mol by 1-C_5_Me_4_, 105 kg/mol by 2-Ind, 92.7 kg/mol by 3-OEt, 98.8 kg/mol by 4-NMe_2_, 148 kg/mol by 5-OMe, and 280 kg/mol by 6-NC_4_H_8_, respectively.

In addition, the chain structure formed by the n-propyl chain formed after the insertion of a molecule of ethylene catalyzed by the insertion of a molecule of 1-octene was considered, and the Gibbs free energy barrier of the β-H elimination reaction was calculated. The calculation results are shown in Table S6 (shown in the Supplementary Materials S2) and correlation Figure 2. It can be observed that when considering the chain structure formed after 1-octene insertion, there is no correlation between the energy barrier of the β-H elimination reaction and the molecular weight of the product, and 6-NC_4_H_8_ and 5-OMe with high molecular weight of the product do not show high β-H elimination reaction energy barrier value.

### 3.2. β-H Transfer to Monomer

**Figure 3:**
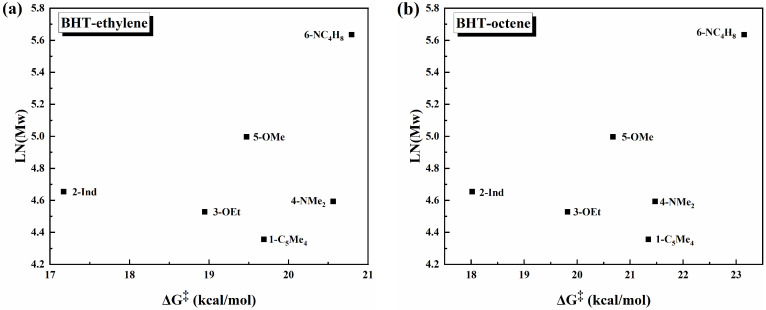
Correlation between the molecular weight (Mw) and the energy barriers (∆G^‡^) of β-H transfer to monomers for n-propyl chain, (**a**) β-H transfer to ethylene, (**b**) β-H transfer to 1-octene. Molecular weight of polymer is 78 kg/mol by 1-C_5_Me_4_, 105 kg/mol by 2-Ind, 92.7 kg/mol by 3-OEt, 98.8 kg/mol by 4-NMe_2_, 148 kg/mol by 5-OMe, and 280 kg/mol by 6-NC_4_H_8_, respectively.

Firstly, we consider the n-propyl chain structure formed after the insertion of a molecule of ethylene and calculate the reaction energy barriers of β-H transfer to ethylene monomer and 1-octene comonomer, respectively, as shown in Table S7 (shown in the Supplementary Materials S2). It can be seen from the fitting Figure 3 that when the β-H transfer reaction of the n-propyl chain is considered, there is no significant positive correlation between the energy barrier of the reaction to ethylene and 1-octene and the product’s molecular weight. In addition, for these six catalysts, the energy barrier of β-H transfer to ethylene monomer is lower than that of β-H transfer to 1-octene.

**Figure 4:**
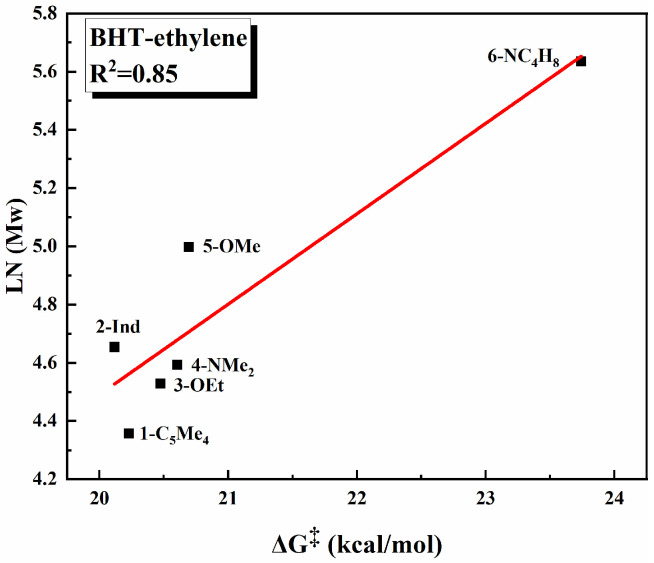
Correlation between the molecular weight (Mw) and the energy barriers (∆G^‡^) of β-H transfer to ethylene for the chain inserted by ethylene and 1-octene. Molecular weight of polymer is 78 kg/mol by 1-C_5_Me_4_, 105 kg/mol by 2-Ind, 92.7 kg/mol by 3-OEt, 98.8 kg/mol by 4-NMe_2_, 148 kg/mol by 5-OMe, and 280 kg/mol by 6-NC_4_H_8_, respectively.

On the other hand, considering that the insertion of 1-octene further catalyzes the n-propyl chain formed after the insertion of an ethylene molecule, the resulting chain structure is hindered by excessive steric hindrance. Therefore, the β-H transfer reaction to monomer only accounts for the transfer to ethylene. The calculated results are depicted in Figure 4. It is evident from the figure that for the β-H transfer to ethylene, there is a correlation between the reaction energy barrier and the product’s molecular weight, with an R^2^ value of 0.85. Additionally, it can be observed that even when 6-NC_4_H_8_ is substituted by tetrahydropyrrole, it still exhibits the highest β-H transfer energy barrier, and the substitution of 5-OMe at the third position of the indene ring also results in relatively high β-H transfer energy barrier compared to 1-C_5_Me_4_, 2-Ind, 3-OEt, and 4-NMe_2_. The 2-Ind catalyst demonstrates the lowest reaction energy barrier, while the other catalysts’ calculated energy barriers are similar.

### 3.3. QSPR Model

A quantitative structure–property relationship model was constructed for the six catalysts discussed above, linking product molecular weight to molecular descriptors to investigate the influence of catalyst ligand structures on the molecular weight of products. The catalyst precursors used in this study were dimethyl compounds. The atomic charges were calculated using the Mulliken and NBO charge calculation methods for Ti, N, Si, and TiMe_2_ groups. Additionally, dipole moments, orbital energies, and relevant geometric parameters were computed, including the distance from the ring center to the central metal, the ring center–central metal-N atom bond angle, and the 3D descriptor buried volume. The associated data are presented in Table S9 of the Supplementary Materials S2. For the selection of buried volume, to comprehensively consider its impact on molecular weight, calculations were performed for different sphere radii and the buried volume values in each quadrant.

**Figure 5:**
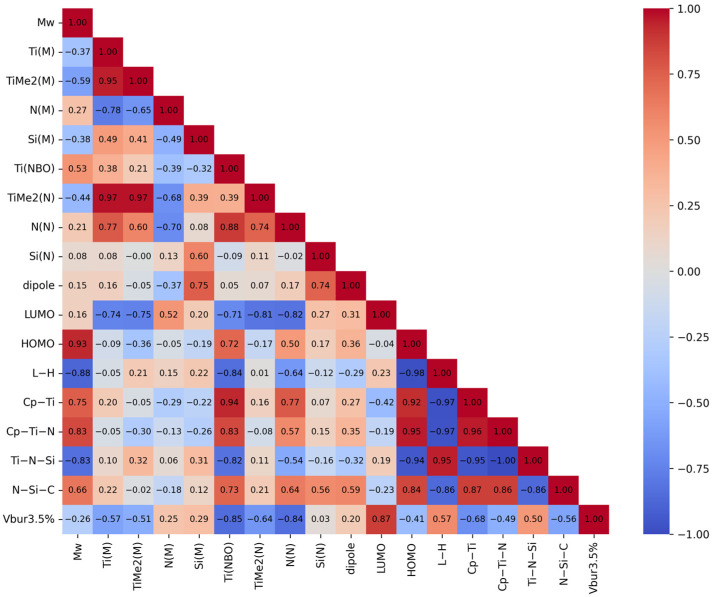
Heat maps displaying the Pearson correlation coefficients between variables.

Firstly, the Pearson correlation coefficients between each descriptor and the molecular weight of the products were calculated, and a heatmap was plotted (Figure 5). The heatmap reveals that the univariate variables showing significant correlations with the molecular weight of the products include the highest occupied orbital energy and related geometric parameters, such as the distance from the ring center to the central metal (Cp-Ti), the ring center–central metal–N atom bond angle (Cp-Ti-N), and the central metal–N atom–Si atom bond angle (Ti-N-Si).

**Figure 6:**
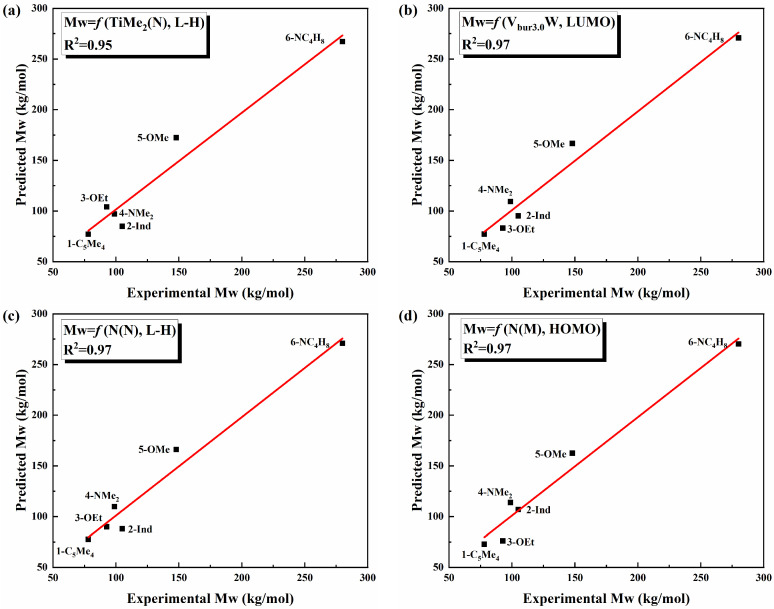
Plots of the molecular weight from experimental data and predicted by QSPR models containing different binary molecular descriptors (**a**) TiMe_2_(N) and L-H, (**b**) V_bur3.0_ and LUMO, (**c**) N(N) and L-H, and (**d**) N(M) and HOMO. Molecular weight of polymer is 78 kg/mol by 1-C_5_Me_4_, 105 kg/mol by 2-Ind, 92.7 kg/mol by 3-OEt, 98.8 kg/mol by 4-NMe_2_, 148 kg/mol by 5-OMe, and 280 kg/mol by 6-NC_4_H_8_, respectively.

Considering the small size of the dataset, only binary linear regression was considered in the QSPR model; the mathematical models are shown in Table S10 of the Supplementary Materials S2. As shown in Figure 6, the experimental values of the molecular weight of the products and the predicted values obtained from the constructed QSPR model are displayed. It can be observed that the binary linear regression model performs well in predicting the molecular weight of the products, with an R^2^ value of 0.95. The molecular feature descriptors that significantly impact the products’ molecular weight include the TiMe_2_ group charge, N atom charge, orbital energy, and the buried volume value.

## 4. Conclusions

For the experimental result of significantly higher product molecular weight in the copolymerization of ethylene and 1-octene catalyzed by CGC with heteroatom substitution, the relationship between chain termination reactions of different structural chains and the product molecular weight was investigated. The Gibbs free energies of the β-H elimination reaction, β-H transfer to ethylene reaction, and β-H transfer to 1-octene reaction for the chain structures formed after ethylene monomer insertion were calculated, as well as the Gibbs free energy barriers for the β-H elimination reaction and β-H transfer to ethylene reaction for the chain structures formed after simultaneous insertion of ethylene and 1-octene. The results indicate a significant positive correlation between the Gibbs free energy barrier of the β-H elimination reaction for the chain structures formed after ethylene monomer insertion and the product molecular weight, with notable correlations for the n-pentyl chain and n-nonyl chain. When considering the Gibbs free energy barrier of the β-H transfer to ethylene reaction for the chain structures formed after the simultaneous insertion of ethylene and 1-octene, there is a positive correlation with the product molecular weight. To provide practical insights for catalyst design, a quantitative structure–activity relationship model was constructed for the product molecular weight and molecular descriptors of these CGCs with heteroatom substitution, showing that molecular feature descriptors such as atomic charges, orbital energy levels, and buried volume have a more significant impact on the product molecular weight. These findings suggest that targeting these molecular features through heteroatom modifications in CGCs could help experimental chemists design catalysts that achieve desired molecular weights in copolymer products.

## Data Availability

The authors do not have permission to share data.

## Supplementary Materials

The following supporting information can be downloaded at: https://www.mdpi.com/article/10.3390/polym16233251/s1, Supplementary Materials S1: Table S1. The energy barriers of β-H elimination for the two conformation of n-pentyl chain. Table S2. The energy barriers of β-H elimination for the two conformation of the chain inserted by ethylene and 1-octene. Table S3. The energy barriers of β-H transfer to ethylene for the two conformation of n-pentyl chain. Table S4. The energy barriers of β-H transfer to ethylene for the two conformation of the chain inserted by ethylene and 1-octene. Figure S1. The two conformations of n-pentyl chain. Figure S2. Transition state structures of β-H elimination for the two conformations of n-pentyl chain. Figure S3. Correlation between the molecular weight and energy barriers of β-H elimination for the two conformation of n-pentyl chain. Figure S4. Correlation between the molecular weight and the final energy barriers of β-H elimination. Figure S5. The two conformations of the chain inserted by ethylene and 1-octene. Figure S6. Transition state structures of β-H elimination of two conformations initiation chains. Figure S7. Correlation between the molecular weight and energy barriers of β-H elimination for the two conformation of the chain inserted by ethylene and 1-octene. Figure S8. Correlation between the molecular weight and the final energy barriers of β-H elimination. Figure S9. Transition state structures of β-H transfer to ethylene for the two conformations of n-pentyl chain. Figure S10. Correlation between the molecular weight and energy barriers of β-H transfer to ethylene for the two conformations of n-pentyl chain. Figure S11. Correlation between the molecular weight and the final energy barriers of β-H transfer to ethylene. Figure S12. Transition state structures of β-H transfer to ethylene for the two conformations of the chain inserted by ethylene and 1-octene. Figure S13. Correlation between the molecular weight and energy barriers of β-H transfer to ethylene for the two conformation of the chain inserted by ethylene and 1-octene. Figure S14. Correlation between the molecular weight and the final energy barriers of β-H transfer to ethylene. Supplementary Materials S2: Table S5. The energy barriers of β-H elimination for the chain inserted by ethylene; Table S6. The energy barriers of β-H elimination for the chain inserted by ethylene and 1-octene; Table S7. The energy barriers of β-H transfer to monomers for n-propyl chain; Table S8. The energy barriers of β-H transfer to ethylene for the chain inserted by ethylene and 1-octene; Table S9. Associated data for QSPR model; Table S10. QSPR models of molecular weights and descriptors.

## References

[1] Li F., Liu W. (2023). Progress in the catalyst for ethylene/α-olefin copolymerization at high temperature. Can. J. Chem. Eng..

[2] Sun M., Xiao Y., Liu K., Yang X., Liu P., Jie S., Hu J., Shi S., Wang Q., Lim K.H. (2023). Synthesis and characterization of polyolefin thermoplastic elastomers: A review. Can. J. Chem. Eng..

[3] Mohite A.S., Rajpurkar Y.D., More A.P. (2022). Bridging the gap between rubbers and plastics: A review on thermoplastic polyolefin elastomers. Polym. Bull..

[4] Ge Y., Hu L., Zhang L., Fu Q., Xu G., Xing Z., Huang L., Zhou W., Chen Y. (2020). Polyolefin elastomer as the anode interfacial layer for improved mechanical and air stabilities in nonfullerene solar cells. ACS Appl. Mater. Interfaces.

[5] Ren Q., Fan J., Zhang Q., Yi J., Feng J. (2016). Toughened polypropylene random copolymer with olefin block copolymer. Mater. Des..

[6] Cano J., Kunz K. (2007). How to synthesize a constrained geometry catalyst (CGC)—A survey. J. Organomet. Chem..

[7] McKnight A.L., Waymouth R.M. (1998). Group 4 *ansa*-Cyclopentadienyl-Amido Catalysts for Olefin Polymerization. Chem. Rev..

[8] Gibson V.C., Spitzmesser S.K. (2003). Advances in non-metallocene olefin polymerization catalysis. Chem. Rev..

[9] Britovsek G.J.P., Gibson V.C., Wass D.F. (1999). The search for new-generation olefin polymerization catalysts: Life beyond metallocenes. Angew. Chem. Int. Ed..

[10] Okuda J. (2003). Rare earth metal complexes that contain linked amido-cyclopentadienyl ligands: Ansa-metallocene mimics and “constrained geometry” catalysts. Dalton Trans..

[11] Okuda J., Musikabhumma K., Sinnema P. (2002). The Kinetic Stability of Cationic Benzyl Titanium Complexes that Contain a Linked Amido-Cyclopentadienyl Ligand: The Influence of the Amido-Substituent on the Ethylene Polymerization Activity of “Constrained Geometry Catalysts”. Isr. J. Chem..

[12] Soga K., Uozumi T., Nakamura S., Toneri T., Teranishi T., Sano T., Arai T., Shiono T. (1996). Structures of polyethylene and copolymers of ethylene with 1-octene and oligoethylene produced with the Cp_2_ZrCl_2_ and [(C_5_Me_4_)SiMe_2_N(t-Bu)]TiCl_2_ catalysts. Macromol. Chem. Phys..

[13] Stevens J.C. (1994). 26. Insite TM Catalysts Structure/Activity Relationships for Olefin Polymerization. Stud. Surf. Sci. Catal..

[14] Klosin J., Kruper W.J., Nickias P.N., Roof G.R., De Waele P., Abboud K.A. (2001). Heteroatom-substituted constrained-geometry complexes. Dramatic substituent effect on catalyst efficiency and polymer molecular weight. Organometallics.

[15] Klosin J., Fontaine P.P., Figueroa R. (2015). Development of group IV molecular catalysts for high temperature ethylene-α-olefin copolymerization reactions. Accounts Chem. Res..

[16] Martínez S., Ramos J., Cruz V.L., Martínez-Salazar J. (2006). Isomeric effect of the Et(H_4_Ind)_2_Zr(CH_3_)_2_ catalyst on the copolymerization of ethylene and styrene: A computational study. J. Polym. Sci. Part A Polym. Chem..

[17] Exposito M., Martínez S., Ramos J., Cruz V., Lopez M., Muñoz-Escalona A., Haider N., Martínez-Salazar J. (2004). Ethylene/styrene copolymerisation by homogeneous metallocene catalysts: Experimental and molecular simulations using rac-ethylenebis(tetrahydroindenyl)MCl_2_ [M=Ti,Zr] systems. Polymer.

[18] Yang S.H., Jo W.H., Noh S.K. (2003). Density functional study of the insertion mechanism for ethylene-styrene copolymerization with constrained geometry catalysts. J. Chem. Phys..

[19] Parveen R., Cundari T.R., Younker J.M., Rodriguez G., McCullough L. (2019). DFT and QSAR studies of ethylene polymerization by zirconocene catalysts. ACS Catal..

[20] Ciancaleoni G., Fraldi N., Cipullo R., Busico V., Macchioni A., Budzelaar P.H.M. (2012). Structure/properties relationship for bis(phenoxyamine)zr(iv)-based olefin polymerization catalysts: A simple dft model to predict catalytic activity. Macromolecules.

[21] Yan M., Kang X., Li S., Xu X., Luo Y., He S., Chen C. (2022). Mechanistic Studies on Nickel-Catalyzed Ethylene Polymerization: Ligand Effects and Quantitative Structure–Activity Relationship Model. Organometallics.

[22] Ratanasak M., Hasegawa J.-Y., Parasuk V. (2021). Design and prediction of high potent*ansa*-zirconocene catalyst for olefin polymerizations: Combined DFT calculations and QSPR approach. New J. Chem..

[23] Zaccaria F., Ehm C., Budzelaar P.H.M., Busico V. (2017). Accurate Prediction of Copolymerization Statistics in Molecular Olefin Polymerization Catalysis: The Role of Entropic, Electronic, and Steric Effects in Catalyst Comonomer Affinity. ACS Catal..

[24] Maity B., Cao Z., Kumawat J., Gupta V., Cavallo L. (2021). A Multivariate linear regression approach to predict ethene/1-olefin copolymerization statistics promoted by group 4 catalysts. ACS Catal..

[25] Uborsky D.V., Mladentsev D.Y., Guzeev B.A., Borisov I.S., Vittoria A., Ehm C., Cipullo R., Hendriksen C., Friederichs N., Busico V. (2020). *C*_1_-Symmetric Si-bridged (2-indenyl)(1-indenyl) *ansa*-metallocenes as efficient ethene/1-hexene copolymerization catalysts. Dalton Trans..

[26] Li Y., Lai X., Xu X., So Y.-M., Du Y., Zhang Z., Pan Y. (2021). Theoretical Study on Ethylene Polymerization Catalyzed by Half-Titanocenes Bearing Different Ancillary Groups. Catalysts.

[27] Lu H., Kang X., Luo Y. (2021). Structure-based relative energy prediction model: A case study of pd(ii)-catalyzed ethylene polymerization and the electronic effect of ancillary ligands. J. Phys. Chem. B.

[28] Liao Z., Du X., Cheng X., Chen Y., Hong X., Zhou S., Yang Y., Li W., Wang J., Yang Y. (2024). Quantitative structure-property relationship study of constrained geometry catalysts for olefin polymerization. New J. Chem..

[29] Frisch M.J., Trucks G.W., Schlegel H.B., Scuseria G.E., Robb M.A., Cheeseman J.R., Scalmani G., Barone V., Petersson G.A., Nakatsuji H. (2016). Gaussian 16, Revision A.03.

[30] Lee C., Yang W., Parr R.G. (1988). Development of the Colle-Salvetti correlation-energy formula into a functional of the electron density. Phys. Rev. B.

[31] Becke A.D. (1993). A new mixing of Hartree–Fock and local density-functional theories. J. Chem. Phys..

[32] Becke A.D. (1988). Density-functional exchange-energy approximation with correct asymptotic behavior. Phys. Rev. A.

[33] Grimme S., Antony J., Ehrlich S., Krieg H. (2010). A consistent and accurate ab initio parametrization of density functional dispersion correction (DFT-D) for the 94 elements H-Pu. J. Chem. Phys..

[34] Zhao Y., Truhlar D.G. (2008). The M06 suite of density functionals for main group thermochemistry, thermochemical kinetics, noncovalent interactions, excited states, and transition elements: Two new functionals and systematic testing of four M06-class functionals and 12 other functionals. Theor. Chem. Acc..

[35] Tomasi J., Mennucci B., Cammi R. (2005). Quantum Mechanical Continuum Solvation Models. Chem. Rev..

[36] (2020). CYLview20; Legault, C.Y. Université de Sherbrooke. http://www.cylview.org.

[37] Falivene L., Cao Z., Petta A., Serra L., Poater A., Oliva R., Scarano V., Cavallo L. (2019). Towards the online computer-aided design of catalytic pockets. Nat. Chem..

