# Supplementary material for: Theoretical Study on the High Polymer Molecular Weight of Heteroatom-Substituted Constrained Geometry Catalyst
Source: Polymers (Basel). 2024 Nov 22;16(23):3251. doi: 10.3390/polym16233251 (PMC11644101; doi:10.3390/polym16233251)
Supplement: Supplementary file 1 [file polymers-16-03251-s001.zip › Supplementary Materials S1.docx]

**Theoretical Study on the High Polymer Molecular Weight of Heteroatom-Substituted Constrained Geometry Catalyst**

Here we discuss the effect of conformation on chain termination reactions, including β-H elimination reaction and β-H transfer to monomer reaction.

**β-H elimination**

When discussing the β-H elimination reaction, the considered transition state structures are all the lowest-energy transition state conformations. To further consider the impact of the conformation of the chain structure inserted by ethylene only and the chain structure inserted by ethylene and 1-octene on the β-H elimination reaction, this part discusses the different conformations of these two chain structures.

For the β-H elimination reaction considering only the chain structure inserted by ethylene, taking into account the degree of correlation with the product molecular weight and the simplicity of the structure, here we choose the n-pentyl chain inserted by two ethylene molecules as the initial chain structure. The two conformations of the n-pentyl chain structure are shown in Figure S1 (taking the unsubstituted 2-Ind catalyst as an example). It can be seen that different conformations of the n-pentyl chain connected to the Ti atom lead to different transition state structures when β-H is transferred to the Ti atom (Figure S2). The relationship between the reaction barriers of β-H elimination under the two conformations of the n-pentyl chain and the product molecular weight is shown in Figure S3. It can be found that the correlation between the reaction barrier of β-H elimination corresponding to conformation 1 and the product molecular weight is stronger.


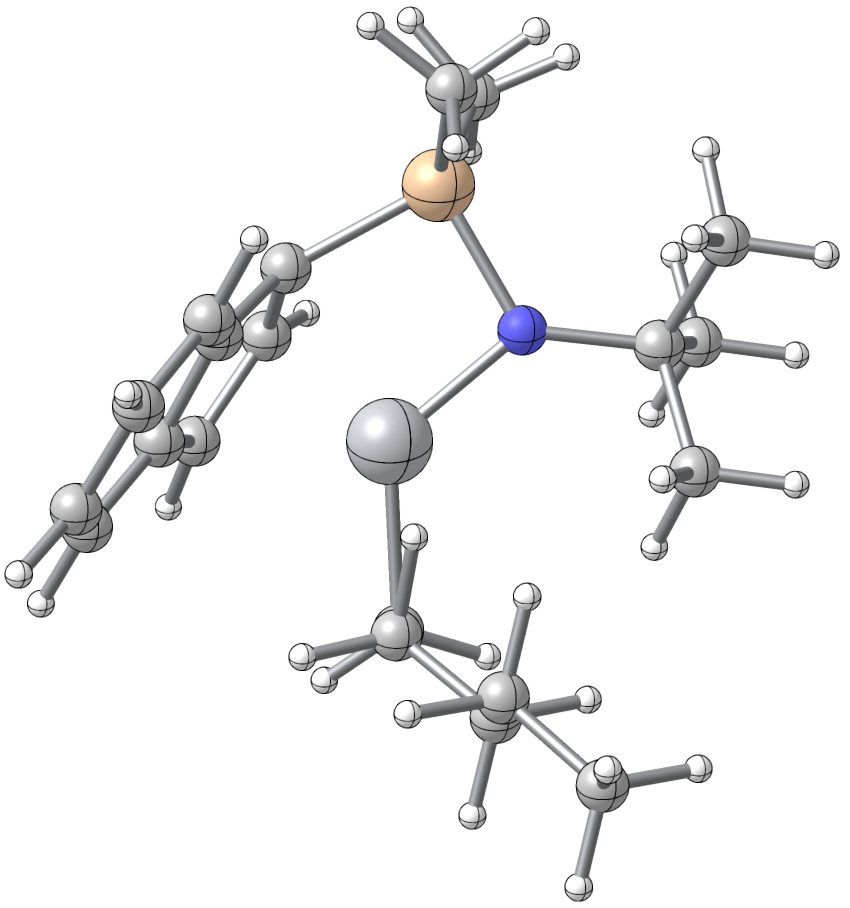

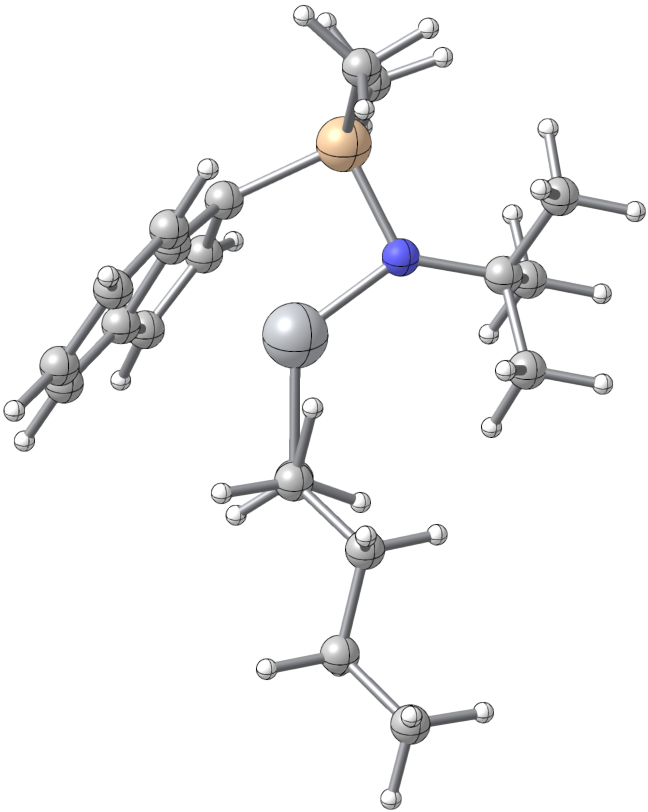


**(b)**

**(a)**

Figure S1. The two conformations of n-pentyl chain, (a) conformation 1, (b) conformation 2.


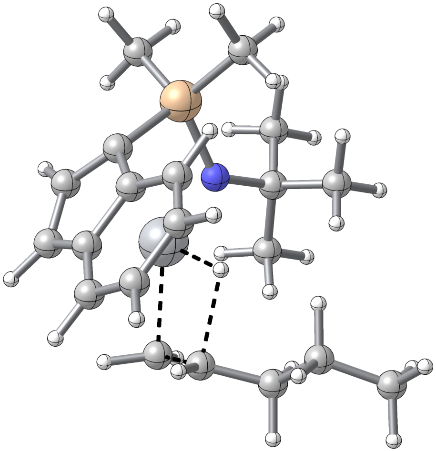

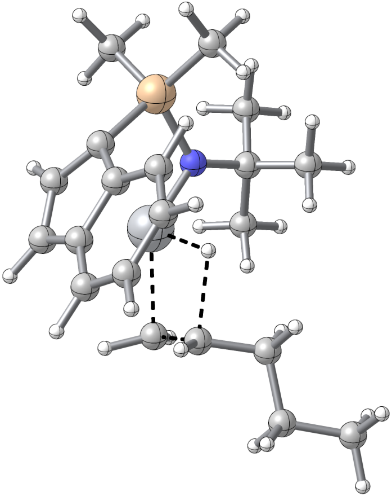


**(a)**

**(b)**

Figure S2. Transition state structures of β-H elimination for the two conformations of n-pentyl chain, (a) conformation 1, (b) conformation 2.

**(b)**

**(a)**


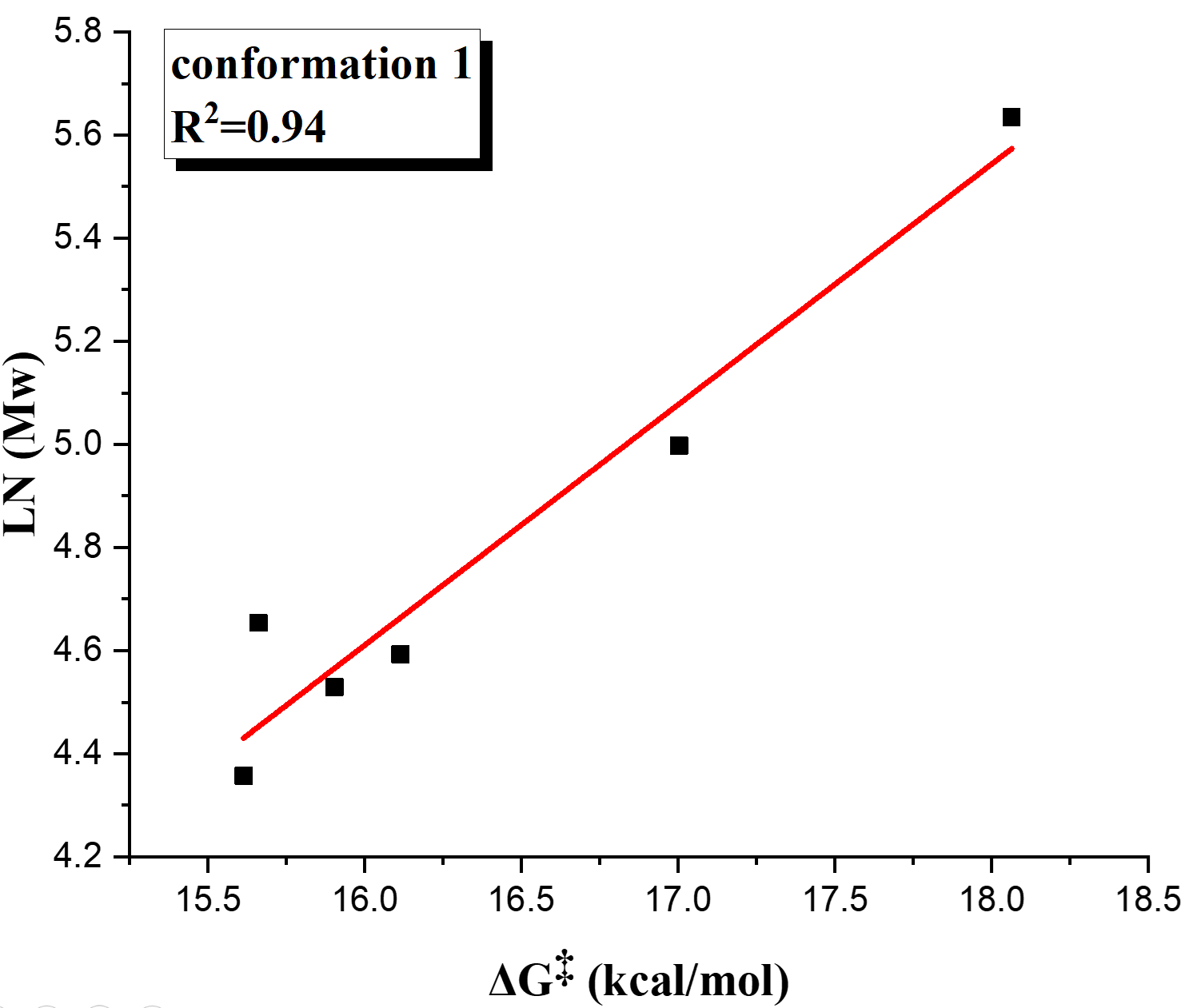

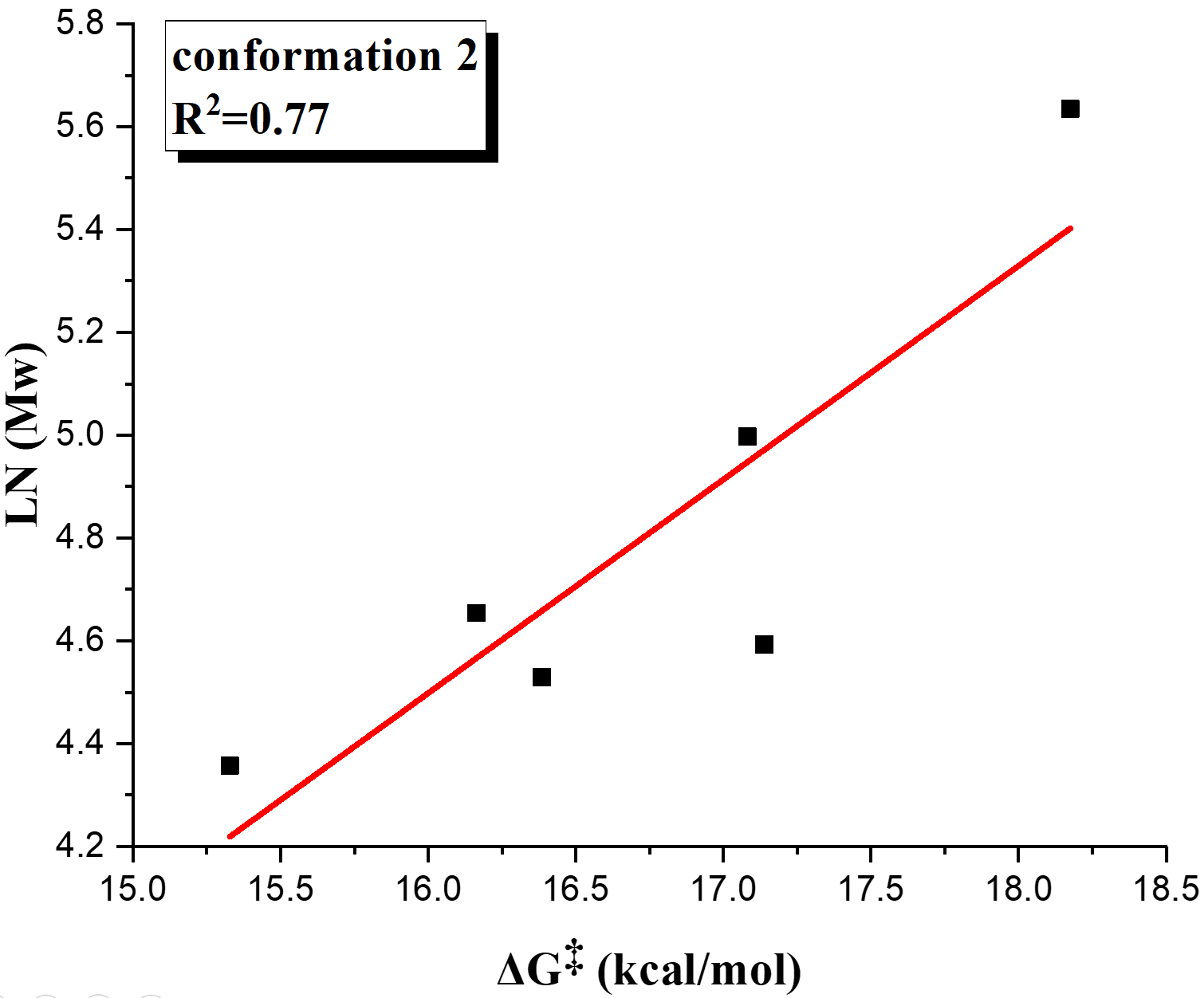


Figure S3. Correlation between the molecular weight and energy barriers of β-H elimination for the two conformation of n-pentyl chain, (a) conformation 1, (b) conformation 2.

For the joint impact of two conformations on the β-H elimination reaction, the Boltzmann distribution of two conformations of the n-pentyl chain is calculated. The calculation formula is as follows:

$$\frac{n_{1}}{n_{2}}=\frac{e^{\frac{{-\triangle G}_{1}^{\ddagger}}{RT}}}{e^{\frac{{-\triangle G}_{2}^{\ddagger}}{RT}}}=e^{\frac{\triangle G_{2}^{\ddagger}}{\triangle G_{1}^{\ddagger}}}$$

Considering the presence of both conformations, the energy barrier of the n-pentyl chain β-H elimination reaction is as follows:

$${\triangle G}_{BHE}=k_{1}*{\triangle G}_{1_{BHE}}+k_{2}*{\triangle G}_{2_{BHE}}$$

Where, $k_{1}$ and $k_{2}$ are the proportions of the two conformations respectively, ${\triangle G}_{1_{BHE}}$ represents the calculated reaction energy barrier of β-H elimination in n-pentyl chain conformation 1, and ${\triangle G}_{2_{BHE}}$ represents the calculated reaction energy barrier of β-H elimination in n-pentyl chain conformation 2, as shown in Table S1. The correlation between the total β-H elimination reaction energy barrier and the molecular weight of the product is shown in Figure S4. Compared with the single conformation (conformation 1: R^2^=0.94, conformation 2: R^2^=0.77), the β-H elimination barrier obtained by considering only the lower transition state Gibbs free energy is more strongly correlated with the molecular weight of the product.

Table S1. The energy barriers of β-H elimination for the two conformation of n-pentyl chain

| Catalyst | 1-Ind | 2-NMe_2_ | 3-OEt | 4-OMe | 5-NC_4_H_8_ | 6-C_5_Me_4_ |
| --- | --- | --- | --- | --- | --- | --- |
| Mw (kg/mol) | 105 | 98.8 | 92.7 | 148 | 280 | 78 |
| Pe1_ΔG^‡^ | 15.66 | 16.11 | 15.90 | 17.00 | 18.06 | 15.61 |
| Pe1_ratio | 63.85% | 50.48% | 59.70% | 64.95% | 66.14% | 71.93% |
| Pe2_ΔG^‡^ | 16.16 | 17.14 | 16.38 | 17.08 | 18.18 | 15.33 |
| Pe2_ratio | 36.15% | 49.52% | 40.30% | 35.05% | 33.86% | 28.07% |
| Pe_ΔG^‡^ | 15.84 | 16.62 | 16.10 | 17.03 | 18.10 | 15.53 |

Where, Pe1 and Pe2 represent conformation 1 and conformation 2 of the n-pentyl chain, respectively, Pe1_ratio and Pe2_ratio represent the percentage of conformation 1 and conformation 2, respectively, and Pe_ΔG^‡^ represents the total β-H elimination energy barrier considering the two n-pentyl conformations, in kcal/mol.

Figure S4. Correlation between the molecular weight and the final energy barriers of β-H elimination.

For the chain structure inserted by ethylene and 1-octene, the two conformations with β-agostic as shown in Figure S5. It can be found that the transition state structure formed during the transfer of β-H to Ti atom is also different due to the difference in the upper branch chain structure of β-C in the two initial chain structures (Figure S6). The correlation between the reaction energy barrier of β-H elimination and the molecular weight of the product under the two initial chain structures is shown in Figure S7, and the expected positive correlation between the reaction energy barrier and the molecular weight of the product is not observed in both conformations.\


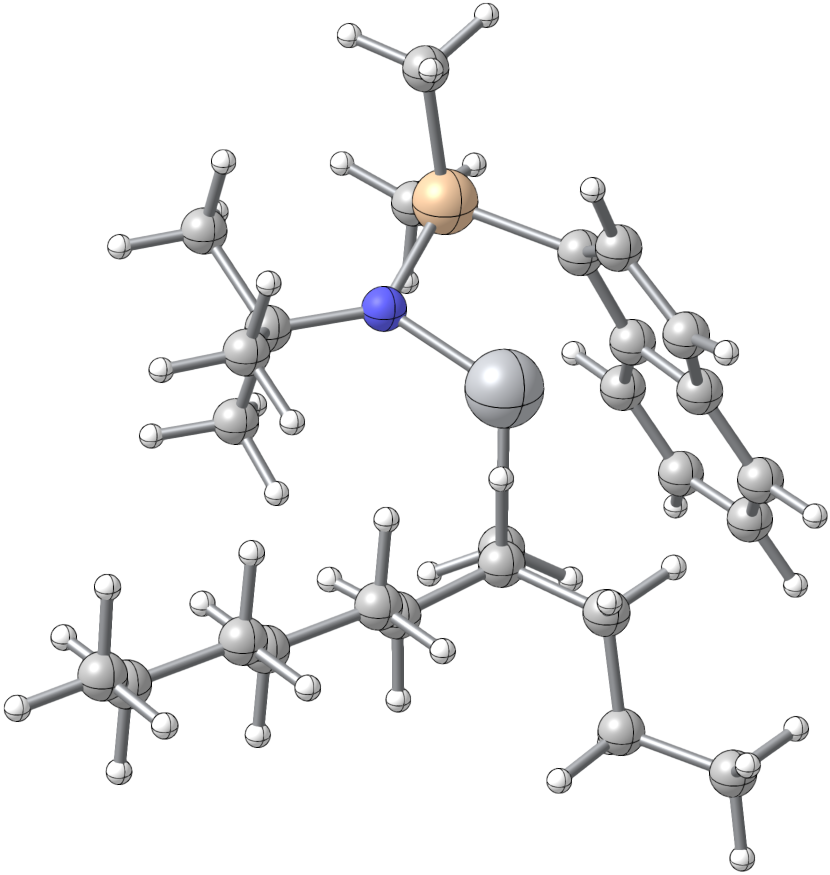

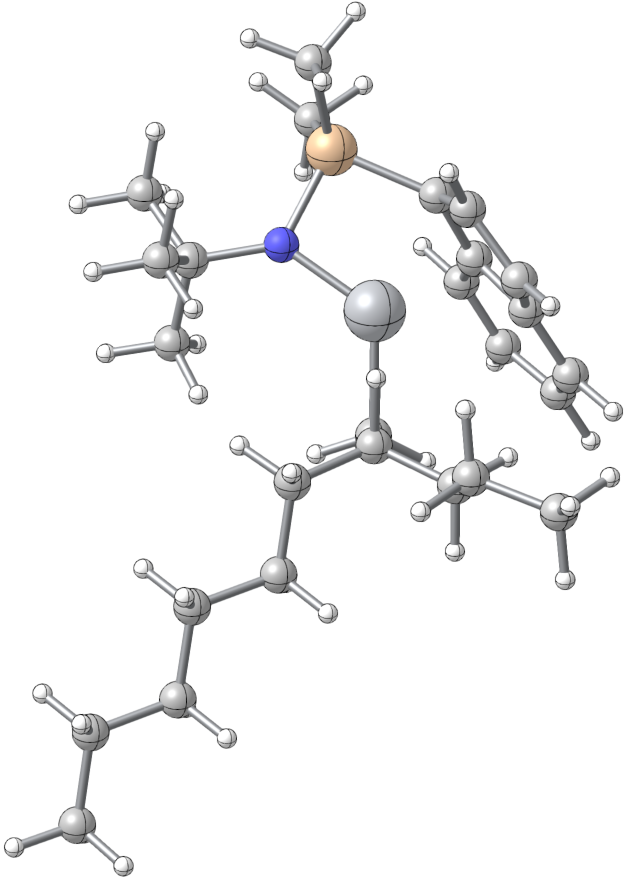


**(b)**

**(a)**

Figure S5. The two conformations of the chain inserted by ethylene and 1-octene, (a) conformation 1, (b) conformation 2.


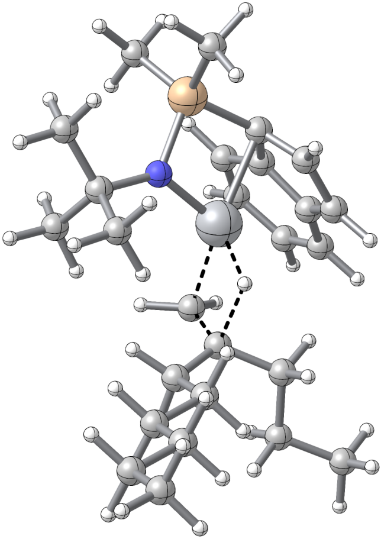

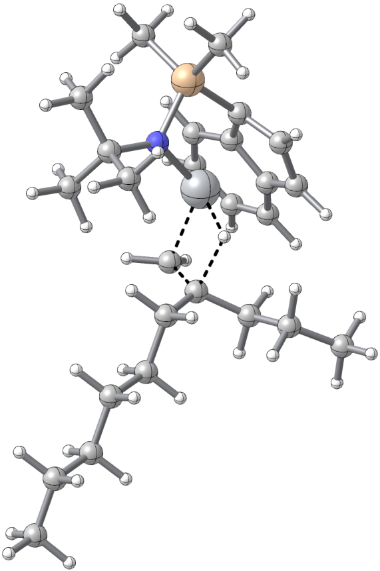


**(b)**

**(a)**

Figure S6. Transition state structures of β-H elimination of two conformations initiation chains, (a) conformation 1, (b) conformation 2.


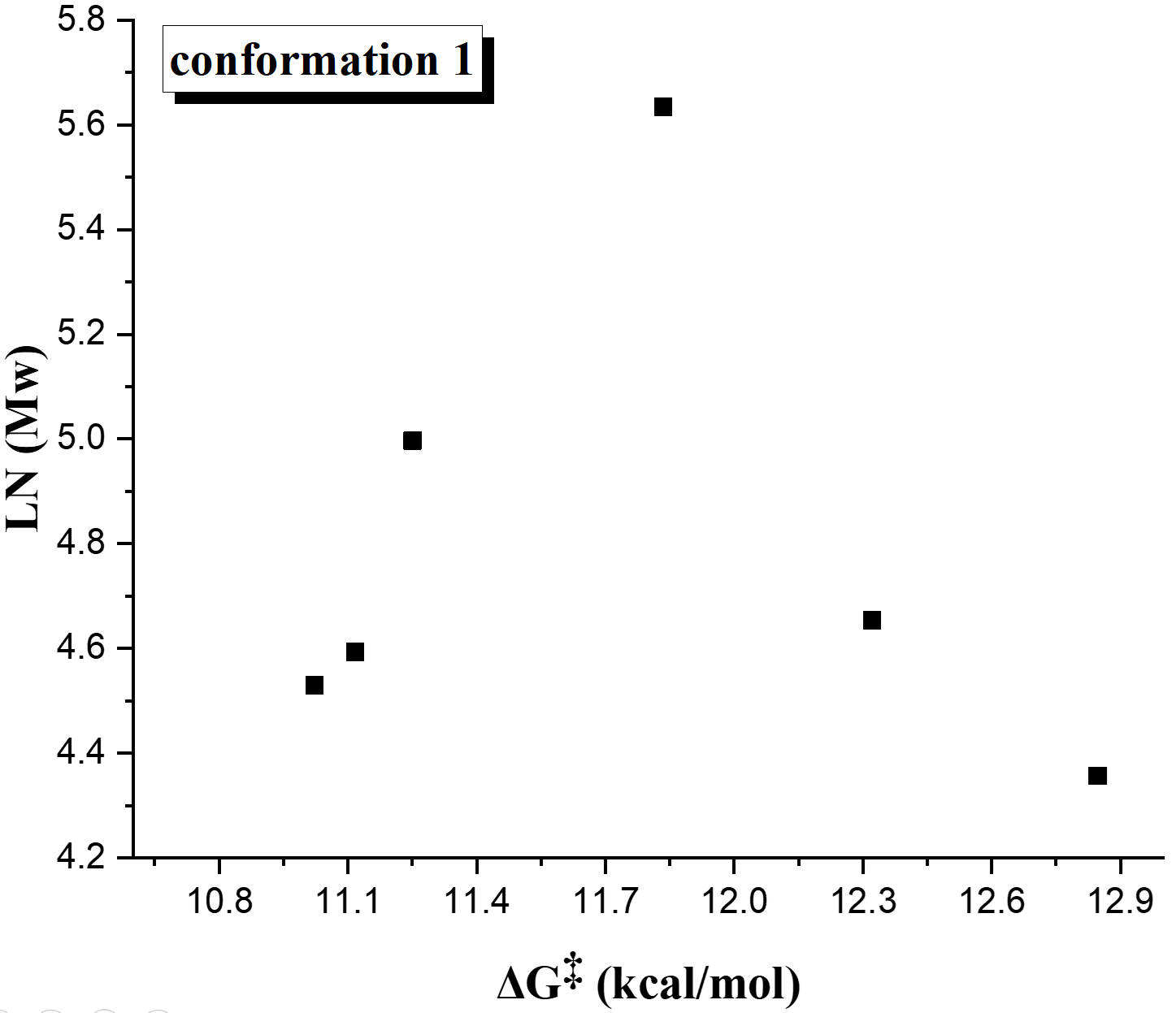

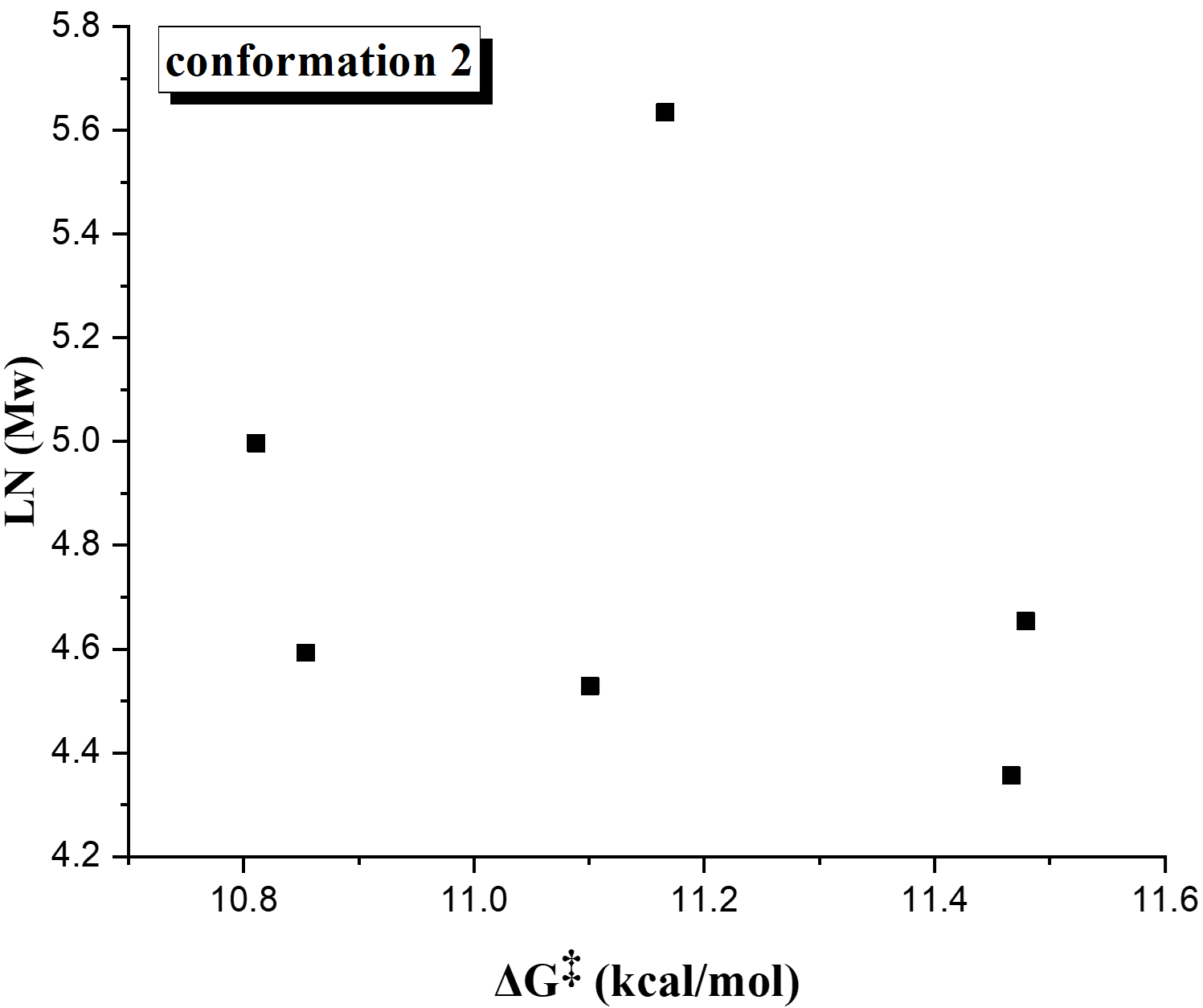


**(b)**

**(a)**

Figure S7. Correlation between the molecular weight and energy barriers of β-H elimination for the two conformation of the chain inserted by ethylene and 1-octene, (a) conformation 1, (b) conformation 2.

For these 6 catalysts, the Gibbs free energy of the initial chain of two conformations was calculated, and the Boltzmann distribution of each conformation was calculated to obtain the final β-H elimination reaction energy barrier, as shown in Table S2. Considering the β-H reaction energy barrier and the molecular weight of the product under the two conformation initiation chains by correlation (Figure S8), it can be observed that there is still no significant correlation between them.

Table S2. The energy barriers of β-H elimination for the two conformation of the chain inserted by ethylene and 1-octene.

| Catalyst | 1-Ind | 2-NMe_2_ | 3-OEt | 4-OMe | 5-NC_4_H_8_ | 6-C_5_Me_4_ |
| --- | --- | --- | --- | --- | --- | --- |
| Mw (kg/mol) | 105 | 98.8 | 92.7 | 148 | 280 | 78 |
| con 1_ΔG^‡^ | 12.32 | 11.12 | 11.02 | 11.25 | 11.83 | 12.85 |
| con 1_ratio | 79.23% | 63.01% | 52.39% | 53.28% | 79.12% | 70.77% |
| con 2_ΔG^‡^ | 11.48 | 10.85 | 11.10 | 10.81 | 11.17 | 11.47 |
| con 2_ratio | 20.77% | 36.99% | 47.61% | 46.72% | 20.88% | 29.23% |
| final_ΔG^‡^ | 12.15 | 11.02 | 11.06 | 11.05 | 11.70 | 12.44 |

Where, con 1 represent conformation 1 of the chain structure inserted by ethylene and 1-octene.

Figure S8. Correlation between the molecular weight and the final energy barriers of β-H elimination.

**β-H transfer to monomer**

This section discusses the influence of initial chain conformation on β-H transfer to monomer reactions, including the n-pentyl initial chain inserted by two ethylene molecules and the initial chain inserted by ethylene and 1-octene.

Firstly, the two conformations of the n-pentyl chain are shown in Figure S1. Since the reaction barriers for β-H transfer to ethylene monomers for all six catalysts are lower than those for β-H transfer to 1-octene comonomers, only the process of β-H transfer to ethylene monomers under the two conformations of the n-pentyl chain is studied in this part. As shown in Figure S9, the transition state structures of β-H transfer to ethylene monomers under the two conformations of the n-pentyl chain both form six-membered ring transition state structures. The relationship between the reaction barriers and product molecular weights for β-H transfer to ethylene under the two conformations of the n-propyl initial chain is shown in Figure S10 It can be found that there is no significant correlation between the reaction barriers and molecular weights under both conformations.


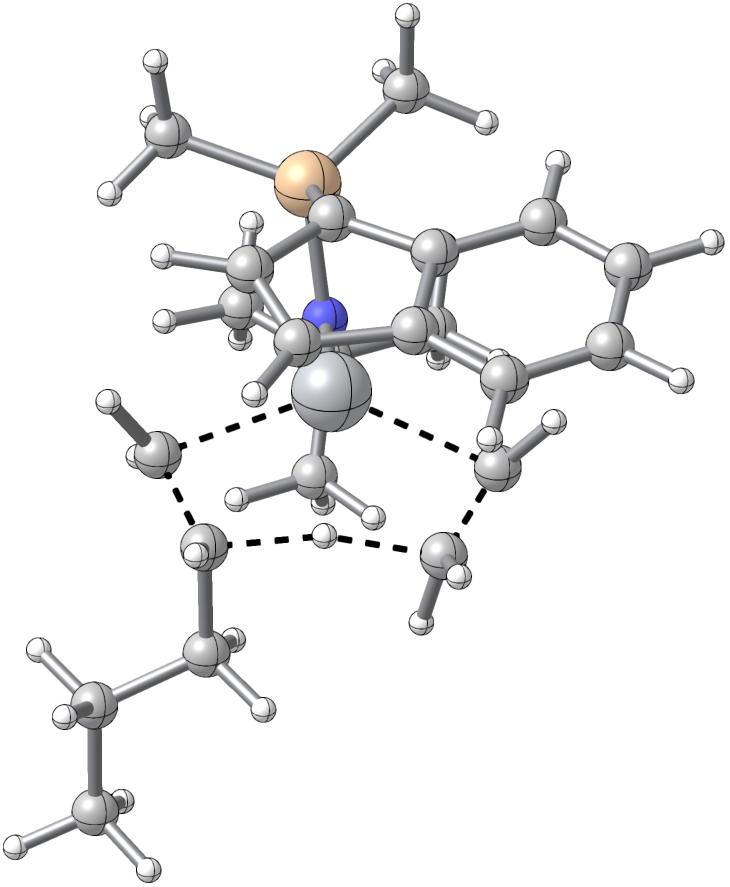

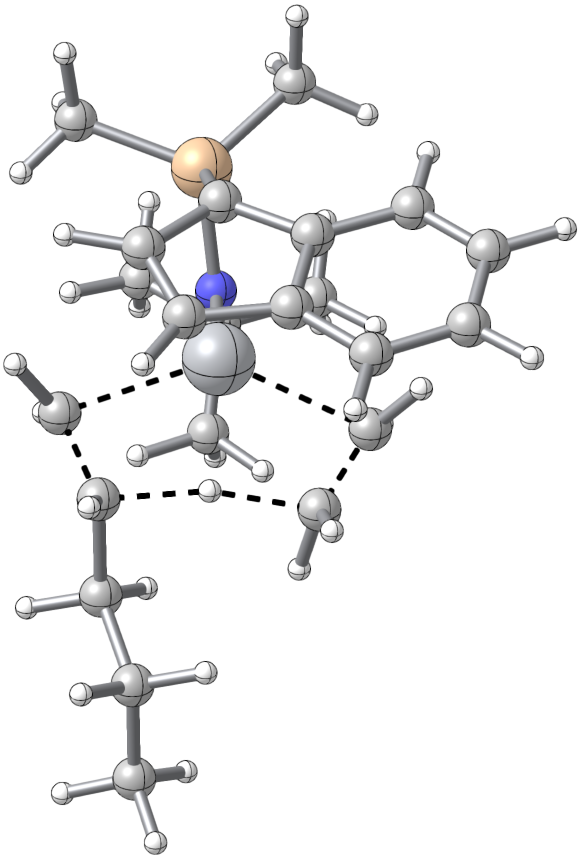


**(a)**

**(b)**

Figure S9. Transition state structures of β-H transfer to ethylene for the two conformations of n-pentyl chain, (a) conformation 1, (b) conformation 2.

**(b)**

**(a)**


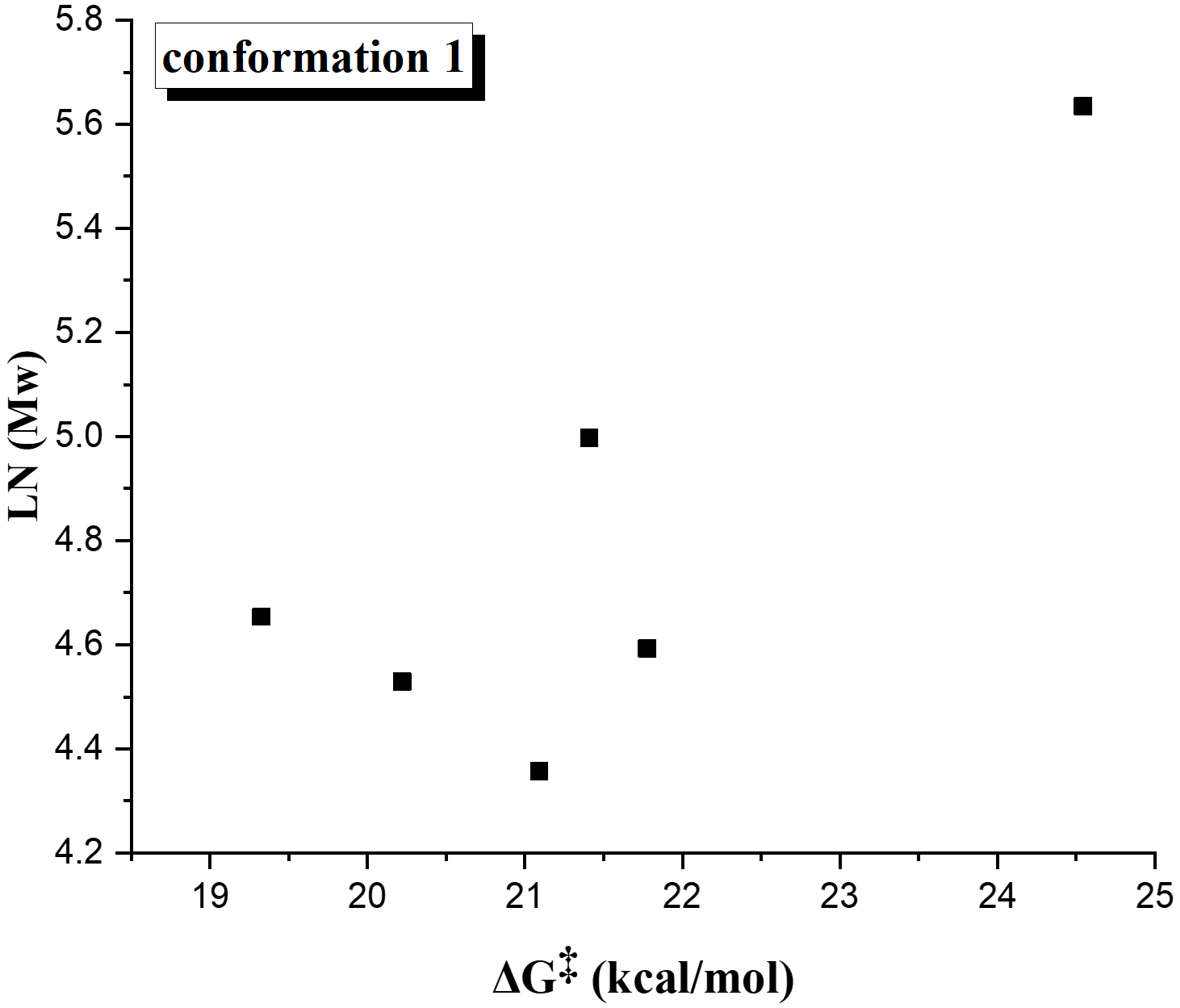

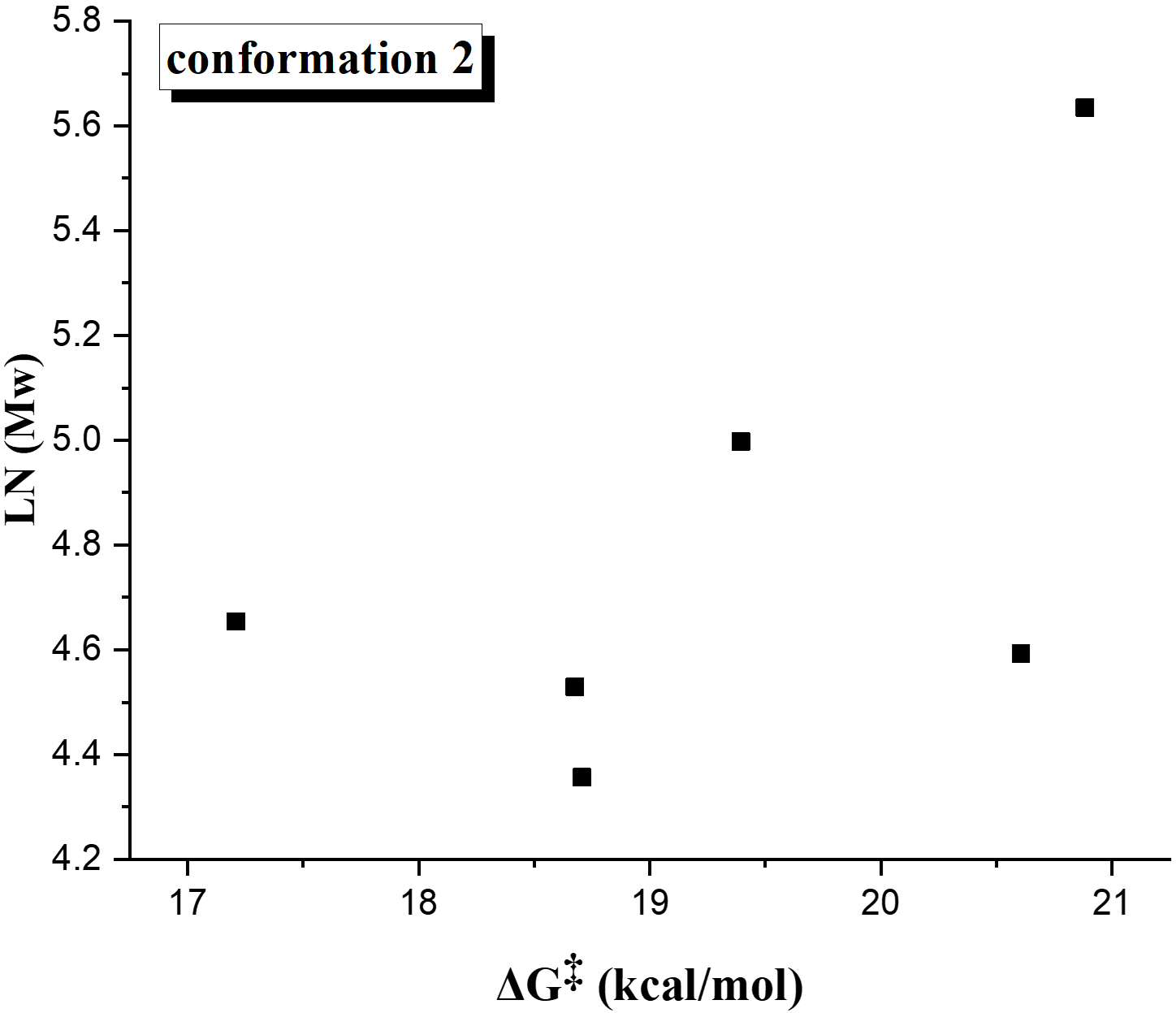


Figure S10. Correlation between the molecular weight and energy barriers of β-H transfer to ethylene for the two conformations of n-pentyl chain, (a) conformation 1, (b) conformation 2.

Similarly, for the two initial conformations of the n-propyl chain, the total β-H transfer barrier to ethylene was calculated using the Boltzmann distribution, as shown in Table S3. The energy barrier and molecular weight of β-H transfer to ethylene monomer in both conformation pentyl chains were correlated. As shown in Figure S11, no significant correlation was observed between the total reaction energy barrier and molecular weight of the product.

Table S3. The energy barriers of β-H transfer to ethylene for the two conformation of n-pentyl chain.

| Catalyst | 1-Ind | 2-NMe_2_ | 3-OEt | 4-OMe | 5-NC_4_H_8_ | 6-C_5_Me_4_ |
| --- | --- | --- | --- | --- | --- | --- |
| Mw (kg/mol) | 105 | 98.8 | 92.7 | 148 | 280 | 78 |
| Pe1_ΔG^‡^ | 19.33 | 21.78 | 20.22 | 21.41 | 24.54 | 21.09 |
| Pe1_ratio | 63.85% | 50.48% | 59.70% | 64.95% | 66.14% | 71.93% |
| Pe2_ΔG^‡^ | 17.21 | 20.60 | 18.68 | 19.39 | 20.88 | 18.71 |
| Pe2_ratio | 36.15% | 49.52% | 40.30% | 35.05% | 33.86% | 28.07% |
| Pe_ΔG^‡^ | 18.56 | 21.20 | 19.60 | 20.70 | 23.30 | 20.42 |

Figure S11. Correlation between the molecular weight and the final energy barriers of β-H transfer to ethylene.

The n-propyl chain formed by the insertion of a molecule of ethylene continues to catalyze the chain structure formed by the insertion of a molecule of 1-octene, and the two conformations of the initial chain are shown in Figure S5. Considering the monomer coordination space around the central metal, the β-H transfer reaction only considers ethylene monomer, and the transition state of the six-membered ring formed by the β-H transfer process of the corresponding two conformations to ethylene is shown in Figure S12. The relationship between the reaction energy barrier of β-H transfer to ethylene in the two conformations and the molecular weight of the product is shown in Figure S13. It can be found that the reaction energy barrier corresponding to the initial chain conformation 1 has a certain correlation with the molecular weight of the product, and the R^2^ is 0.85. No significant correlation was observed between the reaction energy barrier corresponding to conformation 2 and the molecular weight of the product.


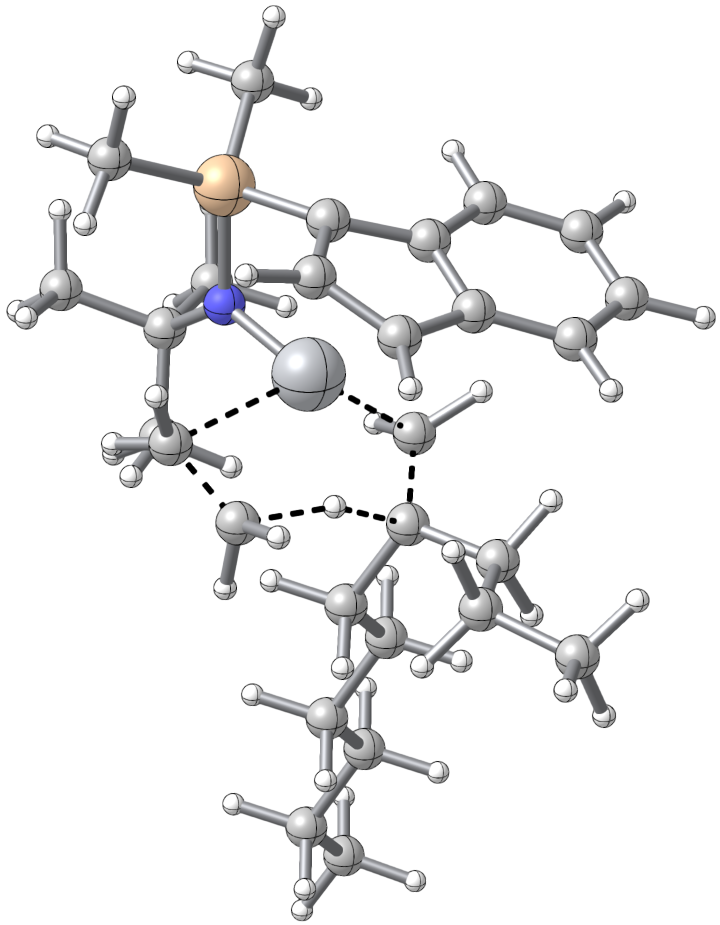

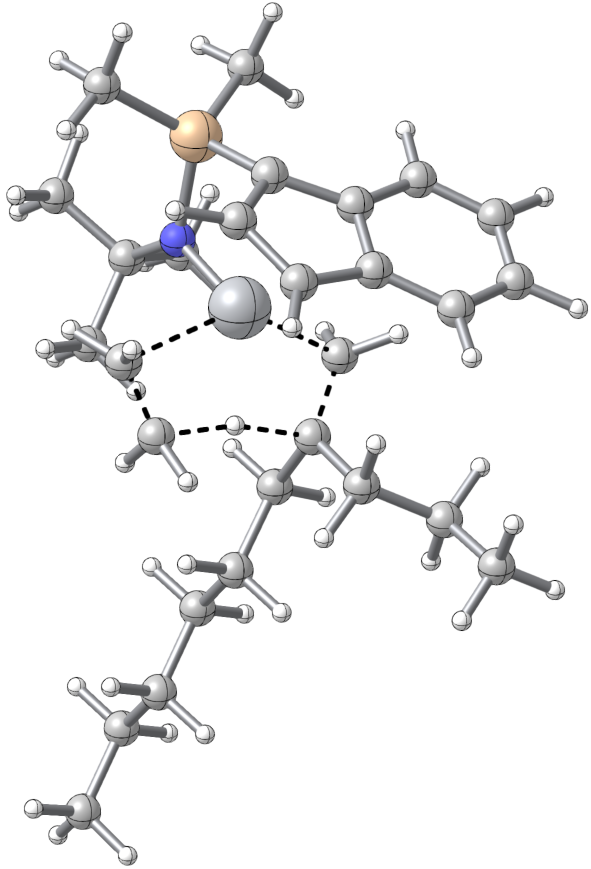


**(a)**

**(b)**

Figure S12. Transition state structures of β-H transfer to ethylene for the two conformations of the chain inserted by ethylene and 1-octene, (a) conformation 1, (b) conformation 2.


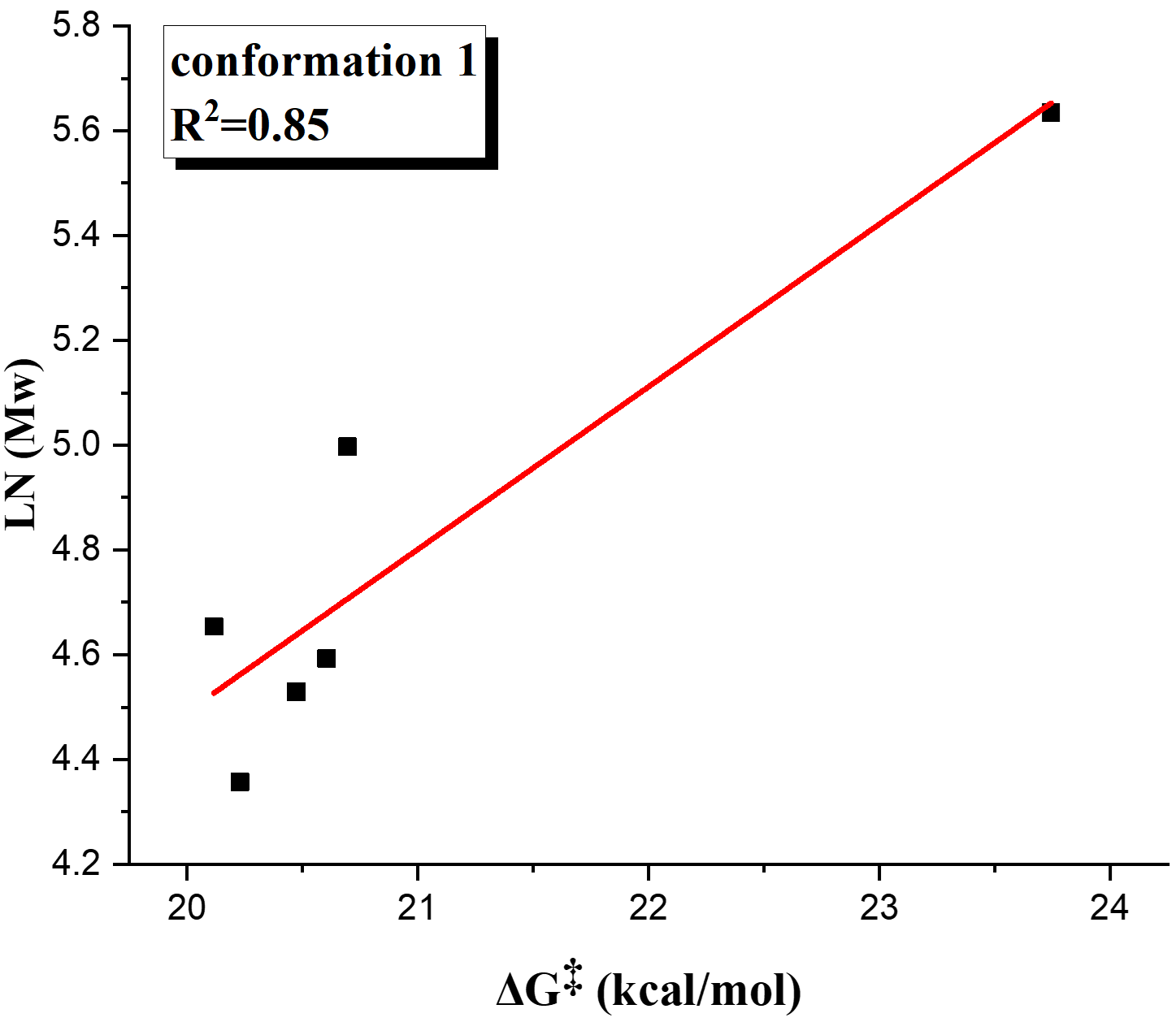

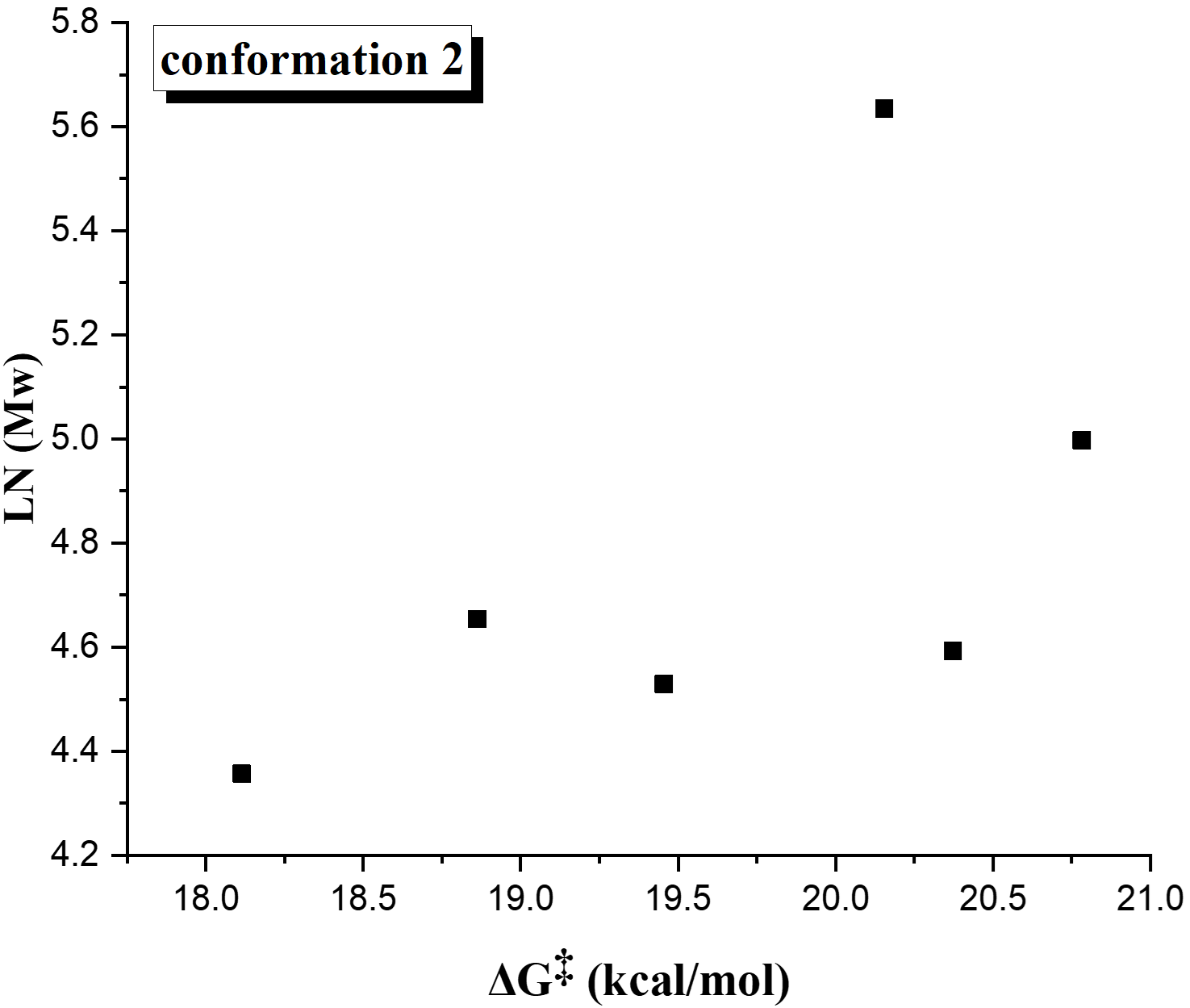


**(b)**

**(a)**

Figure S13. Correlation between the molecular weight and energy barriers of β-H transfer to ethylene for the two conformation of the chain inserted by ethylene and 1-octene, (a) conformation 1, (b) conformation 2.

The Boltzmann distributions of the two chains were used to calculate the total β-H transfer barrier to ethylene, as shown in Table S4. By correlation consideration of the reaction energy barrier of β-H transfer to ethylene in the two conformation initiation chains and the molecular weight of the product (Figure S14), it can be observed that the relationship between the reaction energy barrier and molecular weight of the product is more significantly positive correlation between the total reaction energy barrier and the molecular weight of the product when both conformations are considered separately. Its R^2^ reached 0.93.

Table S4. The energy barriers of β-H transfer to ethylene for the two conformation of the chain inserted by ethylene and 1-octene.

| Catalyst | 1-Ind | 2-NMe_2_ | 3-OEt | 4-OMe | 5-NC_4_H_8_ | 6-C_5_Me_4_ |
| --- | --- | --- | --- | --- | --- | --- |
| Mw (kg/mol) | 105 | 98.8 | 92.7 | 148 | 280 | 78 |
| con 1_ΔG^‡^ | 20.12 | 20.61 | 20.47 | 20.70 | 23.74 | 20.23 |
| con 1_ratio | 79.23% | 63.01% | 52.39% | 53.28% | 79.12% | 70.77% |
| con 2_ΔG^‡^ | 18.86 | 20.37 | 19.45 | 20.78 | 20.15 | 18.11 |
| con 2_ratio | 20.77% | 36.99% | 47.61% | 46.72% | 20.88% | 29.23% |
| final_ΔG^‡^ | 19.86 | 20.52 | 19.99 | 20.74 | 22.99 | 19.61 |

Where, con 1 represent conformation 1 of the chain structure inserted by ethylene and 1-octene.

Figure S14. Correlation between the molecular weight and the final energy barriers of β-H transfer to ethylene.
